# Parent-child relationship of directly measured physical activity

**DOI:** 10.1186/1479-5868-8-17

**Published:** 2011-03-08

**Authors:** Bernard F Fuemmeler, Cheryl B Anderson, Louise C Mâsse

**Affiliations:** 1 Duke University Medical Center, Department of Community and Family Medicine, Durham, NC, USA; 2 Baylor College of Medicine, Department of Pediatrics, Children's Nutrition Research Center, Houston, TX, USA; 3 University of British Columbia, Department of Pediatrics and School of Population and Public Health, Vancouver, BC, Canada

## Abstract

**Background:**

Studies on parent-child correlations of physical activity have been mixed. Few studies have examined concurrent temporal patterns of physical activity and sedentary behaviors in parents and children using direct measures. The purpose of this study was to examine parent-child activity correlations by gender, day of week, and time of day, using accelerometers - a method for direct assessment of physical activity.

**Methods:**

Accelerometers were used to assess physical activity and sedentary time in 45 fathers, 45 mothers and their children (23 boys, 22 girls, mean age 9.9 years) over the course of 4 days (Thursday - Sunday). Participants were instructed to wear accelerometers for 24 hours per day. Data from accelerometers were aggregated into waking hours on weekdays and weekends (6:00 am to midnight) and weekday after-school hours (3:00 - 7:00 pm).

**Results:**

Across the 4 days, the mean minutes per day of moderate-to-vigorous physical activity (MVPA) for fathers was 30.0 (s.d. = 17.3), for mothers was 30.1 (s.d. = 20.1) and for children was 145.47 (s.d. = 51.64). Mothers' and fathers' minutes of MVPA and minutes of sedentary time were positively correlated with child physical activity and sedentary time (all ps < .05, with the exception of mothers' and children's sedentary time on weekdays from 6 am to 12 am). Multivariate linear regression analyses resulted in significant effects between parents and children for MVPA across all time segments. For sedentary activity, significant associations were observed only between father and child on the weekend. Sedentary activity of parents and children were not related for other time segments. Models examining the associations of one or two parents with high levels of MVPA or sedentary time indicated a dose response increase in child activity relative to parent.

**Conclusions:**

Greater parental MVPA was associated with increased child MVPA. In addition, having two parents with higher levels of MVPA was associated with greater levels of activity in children. Sedentary time in children was not as strongly correlated with that of their parents. Findings lend support to the notion that to increase childhood activity levels it may be fruitful to improve physical activity among parents.

## Background

The high rates of obesity among children in the U.S., and globally, are a significant public health concern [1,2]. Although the causes for obesity in society are multifactorial, minimal physical activity, high levels of sedentary time, and excess consumption of energy dense foods are lifestyle factors believed to be contributing to weight gain and risk of obesity in youth [3,4]. Reducing time spent in sedentary activity and increasing moderate-to-vigorous activity (MVPA) has numerous benefits to children's physical and psychological health, including being a promising strategy to prevent obesity in children. Yet, large percentages of children do not meet recommended and optimal levels of regular physical activity [5].

Parents may exert a great degree of influence on their children's physical activity through genetic influence [6] and social learning [7,8]. Within the realm of social learning, parents can serve as role models, encourage their children, or may instrumentally support their children's activity by taking them to events where they can be active [9,10]. The extent to which parents and their children have similar patterns of physical activity levels has been the subject of an increasing body of research because such information would be useful to intervention development [9]. Previous studies have shown that families tend to aggregate on activity patterns, especially in the extremes (e.g., sedentary or vigorous activity) [11-13]. Other studies, however, have not supported parent-child covariation of leisure-time activity [5,14]. A review on this subject by Gustafson and Rhodes (2006) concluded that the results of extant studies on parent-child physical activity (or inactivity) correlations are largely mixed [9]. A possible explanation for this equivocality is that many studies have relied on self-report or parent-report measures rather than more direct measures of physical activity, such as accelerometers [15]. A recent study of children in the UK, found that accelerometer derived parent-child sedentary activity was significantly correlated, but moderate-vigorous activity was not [15]. However, others using accelerometer derived measures of physical activity have found that parents' physical activity levels predict those of their children [16]. In sum, although the use of accelerometers for assessing parent-child correlation in physical activity has been increasing, there are very few studies using this more robust methodology for determining parent-child correlations of physical activity [12,15-17].

There are a number of relevant correlates of children's physical activity engagement [9,18]. A well documented relationship exists between less physical activity engagement and older age [18]. Although among young children (2-5 years), this association appears to be the reverse [19]. In general, girls of any age have lower levels of physical activity than boys [18]. The relationship between race/ethnicity in children and physical activity is not always consistent. Some studies indicate less physical activity engagement among children of racial/ethnic minority groups (especially among girls) [20-22], whereas others indicate greater physical activity or no differences [23,24]. Similarly, mixed findings have also been reported with respect to the association between socio-economic status and physical activity in children; however, higher maternal education and family income appear to be related to greater physical activity engagement, especially among older children [25,26]. Adiposity and overweight status have also been shown to be inversely related to physical activity [27-29], but this is not always found [19]. Finally, although relatively understudied, lower paternal BMI has been reported to be associated with greater physical activity; however, this was observed among boys who were obese [30].

In addition to predictors of childhood physical activity engagement, other factors can be potentially relevant to the parent-child physical activity correlation. For instance, observational studies have shown that daily patterns of activities differ between weekends and weekdays, with less sedentary and more active behavior on weekdays versus weekends among both children and adolescents [31,32] and adults [33]. More fine grained analyses have shown that time of day (e.g., late afternoons) may be an important factor in determining when adolescents are more active [31,34]. Thus, it is reasonable to suspect that the parent-child physical activity relationship could vary with respect to day of the week or time of day. Another potential influential factor on parent-child physical activity correlations could be the gender of either the parent or child. Boys tend to receive more parental support for physical activity than girls [9]. At least one study has found that having physically active parents is more strongly associated with physical activity among boys compared to girls [17]. Patterns of gender-related differences have received little attention in the extant literature [9,35].

The purpose of this study was to measure activity patterns using accelerometers to determine the degree to which physical activity and sedentary time correlate among parents and children. We examined correlations during the weekend, weekday and late afternoon weekday time periods. Associations were further examined using multivariate linear regression models, which included a number of potential covariates of interest. In addition, an exploratory aim examined the overall effect of having sedentary or active parents on their children's overall activity level and if this varied by gender of the child.

## Methods

### Participants

A sub-sample of 57 parent-child triads were recruited from a larger measurement validation study of families with children in 4^th ^and 5^th ^grade from 12 elementary schools in the Southwest US. The current sample provided additional data for the primary study, which developed a measure of parental beliefs about child physical activity [36]. Participants agreed to wear accelerometers 24 hrs per day for 4 consecutive days (Thursday through Sunday). Most of the data were collected in months from August until March when the weather in the Southwest U.S. was temperate. Twelve families were excluded from the analysis: Data from 1 family was excluded due to a malfunctioning accelerometer; 3 families had at least one member with less than 4 valid days of data; 5 families were excluded because only one parent participated, and 3 families were excluded because a member from the "parental" dyad was not an actual parent, but a relative. Complete data on the 4 consecutive days (Thursday - Sunday) for 45 families was available for the analyses (23 boys and 22 girls of 45 parent dyads). The Baylor College of Medicine Institutional Review Board approved this study, and written informed parental consent and child assent was obtained for all participants.

### Measures and Procedures

The MTI Actigraph accelerometer (model 7164; Manufacturing Technologies Inc., Fort Walton Beach FL) was used to objectively measure physical activity. The monitors were set to capture data at 60 second epochs. Actigraph has been shown to provide valid and reliable estimates of physical activity in both adults [37,38] and children [39-41]. Actigraphs were worn on the waist above the right hip using an elastic belt. They were placed on children and their parents on Wednesday afternoon, usually at the participants' home, and removed the following Monday by staff. Participants were instructed to wear the Actigraph continuously during the 24 hour day, except while bathing or swimming for 4 consecutive days. Fathers, mothers, and children wore the Actigraph for approximately 90% of the wear time (96 hr).

### Data Reduction

A SAS program was modified slightly from its original use to read downloaded Actigraph data and produce the necessary outcomes [42]. The data was reduced to include waking hours between 6 am and 12 am. During this 18 hour period of observation the average median hours over the 4 days of observation that children wore the monitor was 17 hours (mean = 17 hours, s.d. = .5 hours). The median hours that fathers wore the monitor was 17.4 hours (mean = 17, s.d. = .9 hours) and the median hours that mothers wore the monitor was 17.3 hours (mean = 17 hours, s.d. = 1.2 hours). The algorithm used 20 minute blocks of consecutive zero counts to identify the non-wear time on a given day. A day was considered valid if the participant wore the accelerometer for at least 10 hours between 6 am and 12 am. Child-specific cut-points [43] and adult cut-points [38] were used to categorize physical activity into minutes spent in the outcome categories of interest, namely Sedentary (< 1.5 METs) and MVPA (> 3 METs). Light METs (1.5 - 3 METs) were not included in the analyses. The decision to merge moderate and vigorous categories was made due to low levels of vigorous activity in the sample. As mentioned above, this study focused on weekend, weekday, and late afternoons (3 pm to 7 pm) and thus data were segmented accordingly and mean minutes of activity per time period were calculated for each of these intervals. To evaluate the overall effect of parents' activity level on children's activity level, the mean minutes of MVPA or sedentary time per day for the 18-hour day (6 am to 12 am) were also examined.

### Data Analysis

The initial analyses included summary statistics of means, standard deviations and ranges of minutes per hour spent sedentary and in MVPA for time segments of weekends, weekdays, and weekday afternoons (3 pm - 7 pm). Bivariate correlations were conducted examining the association between the physical activity level of mothers, fathers, and children (daughters and sons) for the selected time segments. Initial adjusted models using linear regression analyses were performed to examine the effect of mothers' and fathers' sedentary and MVPA on children's sedentary and MVPA. Models included a number of covariates and potential confounders, including maternal and paternal educational attainment, child age, gender, BMI (of children and parents), minority status, and accelerometer wear time. Preliminary analyses showed that child age and minority status were related to at least one of the MVPA and sedentary time outcomes. In general, older age was related to lower MVPA and higher sedentary time on the weekend (MVPA on the weekend = -.42, MVPA on weekday = -.37, MVPA on weekday 3 pm - 7 pm = -.34, and Sedentary on weekend = .39, all ps < .05) and compared to children in a minority ethnic group, Caucasians attained significantly greater MVPA on the weekday (160.2 vs. 117.3) and significantly lower Sedentary activity on the weekend (643.7 vs. 706.0). Thus, these variables were entered as covariates in the adjusted linear regression models. Although accelerometer wear time was fairly uniform, it was associated with sedentary time on the weekend only (r = .51, p < .05), and thus, it was also included in linear regression models. There were no significant associations between activity levels and either mothers' or children's BMI. Fathers' BMI was correlated with children's MVPA on the weekends (r = .33, p < .05). The educational attainment of parents (college graduate or higher versus not having graduated college) was unrelated to MVPA or Sedentary activity among the children. However, because BMI and educational attainment have been found to be relevant to child physical activity in other samples they were included in the linear regression models. Finally, using the observed minutes of MVPA or sedentary time across all days, we evaluated the combined influence of parent activity levels (i.e., having one or two parents who are active or sedentary) on their children's MVPA and sedentary time. To do this, we created categorical variables from parents' MVPA and sedentary time based on a median split. The categorical variable then became 1) having both parents in the low category of MVPA (or low sedentary), 2) at least one parent in the high category of MVPA (or high sedentary), or 3) having two parents in the high MVPA category (or high sedentary). A 3 (both low, one high, both high) by 2 (child gender) Analysis of Variance (ANOVA) was performed to evaluate the main effect of category of exposure to one or two parents, child gender, and the interaction.

## Results

Characteristics of the sample are presented in Table 1. Participants were approximately 70% white and 30% minority, and represented a fairly homogenous sample of medium to high socio-economic status. Table 2 presents the means for minutes per day of MVPA and sedentary, as well as mean counts per minute, for weekends, weekdays, and weekday afternoon time periods. Across the 4 days, the mean minutes per day of MVPA for fathers was 30.0 (s.d. = 17.3), for mothers was 30.1 (s.d. = 20.1) and children was 145.47 (s.d. = 51.64). The mean minutes per day of sedentary time was 769.9 (s.d. = 90.1) for fathers, 739.9 (s.d. = 86.9) for mothers, and 654.4 (s.d. = 77.7) for children.

**Table 1:** Sample characteristics (percentages or means and standard deviations)

Parent Variables	Mothers (n = 45)	Fathers (n = 45)
	**% or Mean (sd)**	

Mean age in years (sd)	40.6 (5.6)	42.8 (6.2)
Education Level		
Less than High School	0	4
High School or Equivalent	4	4
Some College	9	13
College Graduate	44	31
Post Graduate Professional Degree	42	47
Marital Status		
Never married	7	4
Married	91	93
Separated/Divorced/Widowed	2	2
Race		
Hispanic	11	9
Black	4	4
White	71	73
Other	13	13
BMI (kg/m^2^)^†^		
Normal	82	44
Overweight	11	49
Obese	7	7
		
Child Variables	Girls (n = 22)	Boys (n = 23)

Mean Age in years (SD)	10.6 (.63)	10.6 (.76)
BMI^†^		
Normal	82	83
At Risk for Overweight (> 85th percentile)	14	13
Overweight (> 95th percentile)	5	4

**Table 2:** Means and standard deviations for minutes per day of MVPA, sedentary and counts/minute for each time segment

	Sedentary		MVPA		Counts	
	
	Mean (s.d.)		Mean (s.d.)		Mean (s.d.)	
Mothers						
Weekend (6 am to 12 am)	746.2	(95.2)	26.3	(20.4)	292.0	(107.4)
Weekday (6 am to 12 am)	733.5	(101.0)	33.8	(25.2)	330.9	(130.9)
Weekday (3 pm to 7 pm)	162.0	(25.5)	7.7	(7.7)	344.7	(152.5)
Fathers						
Weekend (6 am to 12 am)	744.1	(114.3)	29.5	(18.8)	294.9	(100.2)
Weekday (6 am to 12 am)	795.7	(79.3)	30.5	(23.2)	284.3	(114.8)
Weekday (3 pm to 7 pm)	169.1	(31.5)	8.7	(11.4)	345.2	(229.7)
Children (all)						
Weekend (6 am to 12 am)	651.3	(86.8)	141.8	(55.5)	491.0	(214.1)
Weekday (6 am to 12 am)	657.6	(86.2)	149.1	(56.5)	475.0	(178.5)
Weekday (3 pm to 7 pm)	125.5	(29.9)	50.1	(28.0)	672.7	(427.9)
Daughters						
Weekend (6 am to 12 am)	644.5	(93.0)	138.5	(60.1)	486.3	(240.8)
Weekday (6 am to 12 am)	672.0	(77.6)	128.7	(45.5)	407.9	(113.3)
Weekday (3 pm to 7 pm)	126.3	(24.1)	43.5	(22.0)	596.9	(233.8)
Sons						
Weekend (6 am to 12 am)	657.9	(82.0)	145.0	(51.9)	495.6	(190.5)
Weekday (6 am to 12 am)	643.7	(93.3)	168.7	(59.9)	539.2	(206.6)
Weekday (3 pm to 7 pm)	124.8	(35.1)	56.5	(31.9)	771.0	(541.7)

### Correlations between Parents and Children

Bivariate correlations for sedentary and MVPA stratified by child and parent gender are presented in Table 3. According to Cohen (1992), correlation coefficients of .10 are considered small, .30 considered medium, and .50 considered large [44]. Fathers' and sons' MVPA were significantly and positively correlated during the weekend and during the weekday afternoon hours (r = .43 and .55, respectively). Mothers' and sons' MVPA were not significantly correlated during any of the segmented times. Fathers' and daughters' MVPA were significantly correlated during the weekdays (r = .42), but not during the after-school period. However, there was a fairly robust correlation between mothers' and daughters' MVPA for all time segments (r = .67 on the weekends, .70 on weekdays, and .62 for after-school). With regard to sedentary counts, significant correlations were found for sons' during the weekend and after-school period with both of their parents (r = .44 and .46 with mothers' and fathers' on weekends, respectively, and r = .60 and .45 with mothers' and fathers' for after-school, respectively). Daughters' sedentary activity was significantly correlated with mothers' (r = .52) and fathers' (r = .65) during the weekend, and with fathers' during the weekday (r = .61). Partial correlations adjusting for minority status, age, BMI (of children and parents) and educational attainment of both mothers and fathers did not markedly affect the statistical significance for many of the correlations (data not shown). However, the significant correlation between fathers' and daughters' MVPA and sedentary activity during the weekday time period was reduced to non-significance, as was the significant correlations between both parents and sons' sedentary activity on the weekend.

**Table 3:** Bivariate correlations between mothers', fathers' and children's sedentary and MVPA levels

	Sedentary			MVPA		
	**Daughters**	**Sons**	**All**	**Daughters**	**Sons**	**All**

Weekend (6 am to 12 am)						
Mothers	.52*	.44*	.43**	.67**	.10	.45**
Fathers	.65**	.46*	.56**	.37	.43*	.40**
Weekday (6 am to 12 am)						
Mothers	.30	.13	.23	.70**	.09	.39**
Fathers	.61*	.09	.31*	.42*	.38	.41**
Weekday (3 pm to 7 pm)						
Mothers	.19	.60**	.39**	.64**	.13	.34*
Fathers	.33	.45*	.39**	.19	.55**	.46**

* p < .05, ** p < .01

### Linear Regression Analyses

Results of the linear regression analyses represent the relative association of mothers' or fathers' activity (either MVPA or sedentary) with children's, adjusted for minority status, age, gender, BMI (of child and parents), educational attainment (of both parents), and accelerometer wear time (Table 4). Fathers' and mothers' MVPA were each statistically significantly associated with children's MVPA during the weekend (p = .01 and p = .02, respectively), during the afternoon hours (p < .01 and p = .01, respectively), and during the weekday (p = .03 and p = .04, respectively). Results of the linear regression analyses for children's sedentary time (Model 1b) indicated that fathers' sedentary time was significantly associated with their children's during the weekend (p < .01), but not during any of the other time segments.

**Table 4:** Linear Regression Analysis for Variables Predicting Children's Activity Level

Variable	B	SE B	β	p-value	B	SE B	β	p-value	B	SE B	β	p-value
Child MVPA	Weekend (6 am to 12 am)				Weekday (6 am to 12 am)				Weekday (3 pm to 7 pm)			
Model 1a												
Gender (male)	-4.75	15.32	-0.04	0.76	21.60	15.80	0.19	0.18	0.05	6.98	0.00	0.99
Fathers' MVPA	1.03	0.38	0.35	0.01	0.76	0.33	0.31	0.03	1.30	0.29	0.53	0.00
Mothers' MVPA	0.88	0.36	0.32	0.02	0.63	0.30	0.28	0.04	1.29	0.46	0.35	0.01
												
Child Sedentary												
Model 1b												
Gender (male)	39.28	25.85	0.23	0.14	-0.49	32.00	0.00	0.99	5.88	10.46	0.10	0.58
Fathers' Sedentary	0.37	0.12	0.50	0.00	0.19	0.21	0.18	0.38	0.23	0.18	0.24	0.22
Mothers' Sedentary	-0.01	0.16	-0.01	0.96	0.05	0.16	0.06	0.76	0.41	0.23	0.34	0.08

β = standardized beta; Models controlled for minority status, child age, gender, BMI of parents and child, maternal and paternal education, and wear time

### Effect of one or two parents active or inactive

**Figure 1:**
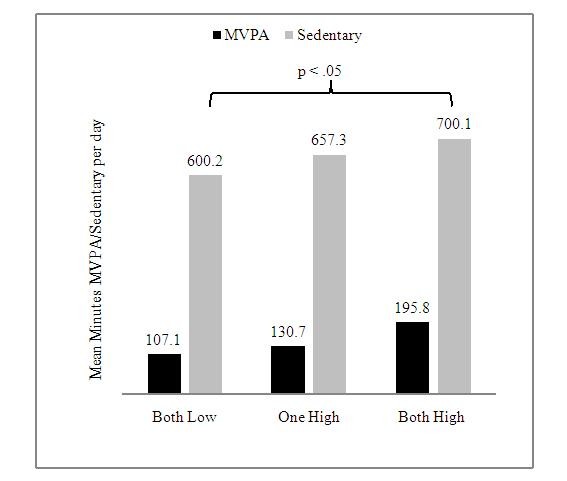
**Mean minutes of MVPA and sedentary time by parental activity status**.

Across all days of monitoring, children's MVPA and sedentary time were subjected to a 3 by 2 ANOVA; 3 parental levels of MVPA (both parents have high levels of MVPA, both parents have low levels of MVPA, and one parent with high levels while the other with low levels of MVPA) and 2 categories for gender. With MVPA among the children as the dependent variable, the main effect of parental MVPA yielded an F ratio of F (2, 44) = 105.48, p < .01, such that the children's MVPA mean was significantly greater with two parents having high levels of MVPA (M = 195.83, SD = 14.38) compared to having both parents with low levels of MVPA (M = 107.09, SD = 10.17) (Figure 1). The mean MVPA for children with only one parent having high levels of MVPA (M = 130.73, SD = 17.62) was not significantly different from the other two parental levels of MVPA. There was no significant main effect for gender F (1, 44) = 0.664, p > .05 and no significant interaction between parental levels of MVPA and gender F (1, 44) = 0.219, p > .05.

An ANOVA for children's sedentary time was performed in a similar fashion as above. Results indicated that the average time children were sedentary was M = 600.19, SD = 20.1 when both parents were low in sedentary, was M = 657. 3, SD = 15.6 when one parent was high in sedentary and the other was low, and was M = 700.11, SD = 19.3 when both parents were high in sedentary (Figure 1). Despite the increasing means relative to increasing parental levels of sedentary time, there was no significant main effect for parental sedentary time F (2, 44) = 4.14, p > .05, gender F (1, 44) = .11, p > .05, or the interaction F (2, 44) = 1.63, p > .05.

## Discussion

To better understand and affect children's physical activity levels, recent research on the determinants of physical activity has expressed the need for studies that more precisely investigate the varying contexts in which parental activity is related to children's activity [9,45]. The data presented in the current study add to the literature examining parent-child correlations of activity by examining gender-related differences as well as examining patterns during relevant weekly segments: weekend, weekday, and weekday after-school hours. The number of studies using accelerometer derived measures of physical activity to assess parent-child correlations in activity has been increasing, and this study is in line with this small, but growing, body of research.

In these data, we found that, overall, MVPA of parents and children were significantly correlated for many of the observed time segments (weekend, weekday, and weekday 3 to 7 pm). These associations remained significant in multivariate regression models. However, the association between parents' and children's sedentary activity was not as consistent. In the regression models, the only statistically significant finding was that fathers' sedentary activity was associated with children's on the weekend. We also found that children of two highly active parents engaged in more MVPA than children of parents who engaged in very little MVPA. The MVPA correlations stratified by gender of parent and child were interesting in that they appear to be gender specific (i.e., mothers' were correlated with daughters' and fathers' with sons'), especially for the weekend and weekday after-school time segments. Greater attention to gender specific association in future studies seems warranted.

Comparing our findings to those few existing studies of parent-child correlations using accelerometer derived measures of physical activity [12,15-17] is difficult because; 1) the age ranges of children differ across studies, 2) some studies had data from only one parent, whereas others had data from both mothers and fathers, and 3) slightly different analytic methods have been employed across studies. However, in general, our findings comport with those that have found that parental physical activity is positively associated with an increase in children's physical activity [16] and those that find that children are more likely to be active when their parents are active [12,17]. Notably, the one other study using accelerometer derived measures of physical activity found parents' sedentary activity was significantly correlated with their children's sedentary activity, but MVPA was not [15].

The study findings suggest several key points relevant to the ongoing efforts to better understand parent-child correlations in physical activity patterns. First, during times when most parents could potentially have the most direct influence on child activity (i.e., after-school and on weekends) both mothers' and fathers' MVPA were positively related to their children's. Although our data does not allow us to know whether parental modeling, support, shared activities, or combinations of such factors were responsible for the parent-child activity aggregation found, it seems clear that time periods outside of work or school are crucial targets for interventions that aim to involve parents.

Second, the results add to the increasing evidence regarding the importance of the after-school hours in youth behavior and health. Higher levels of physical activity have been found in the time period after-school in previous studies [31,32,34], and interventions that have targeted this time period have been successful in increasing physical activity and decreasing overweight in children and adolescents [46-48]. Previous studies have also reported distinct gender differences (boys > girls) in levels of MVPA during the after-school period that were not clearly replicated in our study [31,34]. The boys in our sample had higher MVPA means during the after-school hours than the girls, but they were not statistically significantly higher (data not reported). Importantly, the MVPA levels of parents' were predictive of children's activity after-school, controlling for gender; although the correlations stratified by gender hint that these associations may be gender-specific. This deserves further study in other samples. Interestingly, in multivariate models we did not find that parents' sedentary time during the after-school segment predicted that of their children's.

Third, our study extends findings on the importance of having active parents as a predictor of physical activity in children. Although parental modeling has been well accepted as a possible mechanism for parent-child aggregation of physical activity, there have been very few studies that have looked at the impact of one versus two active parents using an accelerometer derived measure to quantify activity [9]. Similar to findings by Moore et al. [17], we found a strong, linear relationship between the number of active parents and the activity levels of children. Children with two active parents engaged in greater MVPA than children where both parents were low in MVPA. We did not find any child gender effects, as Moore et al. did in their study, in which they found that parental activity was stronger for boys. In our study, in which the children were slightly older, children appeared to benefit substantially more from two active parents and this was true for both boys and girls. With respect to sedentary activity, children's sedentary activity time did increase in a graded fashion with the number of sedentary parents, but these were not statistically significant increases in mean sedentary activity time. In other words, high levels of sedentary time existed for all children, regardless of the sedentary classifications of the parents.

Our data, and the multiple analytical approaches we have used to interpret it, demonstrate the independence of MVPA and sedentary time [49] in parent-child correlations. Biddle and others have argued that even highly active people spend considerable time being sedentary [50], and this may have been the case among our participants. In sum, our data lend support to the notion that to increase childhood activity levels it may be fruitful to focus on improving the MVPA among the whole family, including both parents.

As in any study, our results should be considered with respect to the limitations. First, the sample size was small which may have had an impact on our ability to detect significant associations other than large effects. The sample was also fairly homogenous, especially in regard to parental education and weight status, which limits the degree to which our findings generalize to the broader population. Notably, however, the sample did include a larger percentage of non-white participants than is usually present in these types of studies. Population based studies of parent-child correlations using directly measured physical activity seem warranted. Another limitation is that the study design was cross-sectional. Longitudinal studies of parent-child physical activity correlations are needed especially in light of the well established finding that physical activity levels decline with age [24,51-54]. It remains to be determined how parent-child correlations change through development. Another methodological factor to consider relates the use of accelerometers. Although we believe that accelerometer derived measures of physical activity are useful, their sole use can present some limitations. In general, accelerometers like the type used in this study, have been shown to be a valid method for assessing physical activity in children [55]. However, these devices do not capture certain types of activity well, such as cycling, climbing stairs, or swimming [56]. Further, future studies should be designed to provide more contextual information on what parents and children are doing during time periods of interest (e.g., time use data), which would increase understanding of the types of activities in which parents and children engage and if they are active or inactive together. Lastly, although not necessarily a limitation, the total amount of MVPA was higher in children than in parents. This is in line with the noted observation of declining physical activity with increasing age. This difference in the total amount likely reflects the different ways in which children and adults accumulate MVPA (e.g., spontaneous play vs. structured or planned activities). We were not able to capture that level of detailed information in this study. Future studies are needed to look at *how *parents and children of different ages accumulate their MVPA throughout the day and the mechanisms that help explain the parent-child correlations we observed in this study. Understanding these explanatory factors will be beneficial to interventions that aim to increase children's MVPA through increasing parental MVPA.

## Conclusions

The present study is important and unique in its contribution to the literature examining parent-child correlations in accelerometer derived measures of physical activity. Although it is commonly asserted that parents have a significant influence on their child's adoption of a physically active lifestyle, many previous studies have not used accelerometer derived measures of physical activity nor have they examined how correlations vary with respect to time of day or day of week[9]. As a result, equivocal findings have been reported. The present findings on patterns of activity among parents and children suggest that parents' MVPA is related to their children's and such findings are useful for justifying family-based interventions. Future studies are needed to confirm our findings as well as extend this literature examining parent-child correlations in physical activity.

## Competing interests

The authors declare that they have no competing interests. The authors have no financial arrangement or affiliation with any products or services used or discussed in this paper.

## Authors' contributions

All authors (BFF, CBA, LCM) contributed to the design, analysis, and drafting of the manuscript. Data for this study were originally collected as part of a larger measurement validation study conducted by CBA. All analyses were conducted by BFF in consultation with LCM and CBA. All authors read and approved the final manuscript.
